# Antimicrobial and Antibiofilm Capacity of Chitosan Nanoparticles against Wild Type Strain of *Pseudomonas* sp. Isolated from Milk of Cows Diagnosed with Bovine Mastitis

**DOI:** 10.3390/antibiotics9090551

**Published:** 2020-08-28

**Authors:** Paula Rivera Aguayo, Tamara Bruna Larenas, Carlos Alarcón Godoy, Bernardita Cayupe Rivas, Jorge González-Casanova, Diana Rojas-Gómez, Nelson Caro Fuentes

**Affiliations:** 1 Centro de Investigación Austral Biotech, Facultad de Ciencias, Universidad Santo Tomas, Avenida Ejército 146, Santiago 8370003, Chile; privera@australbiotech.cl (P.R.A.); tbruna@australbiotech.cl (T.B.L.); calarcon@australbiotech.cl (C.A.G.); bcayupe@australbiotech.cl (B.C.R.); 2 Instituto de Ciencias Biomédicas, Facultad de Ciencias de la Salud, Universidad Autónoma de Chile, Santiago 8910060, Chile; jorge.gonzalez@uautonoma.cl; 3 Escuela de Nutrición y Dietética, Facultad de Medicina, Universidad Andres Bello, Santiago 7591538, Chile; diana.rojas@unab.cl

**Keywords:** bovine mastitis, antibiofilm, *Pseudomonas*, chitosan, nanoparticles

## Abstract

Bovine mastitis (BM) is the most prevalent bacterial infection in the livestock sector, affecting the dairy industry greatly. The prevention and treatment of this disease is mainly made via antibiotics, but the increasing antimicrobial resistance of pathogens has affected the efficiency of conventional drugs. *Pseudomonas* sp. is one of the pathogens involved in this infection. The therapeutic rate of cure for this environmental mastitis-causing pathogen is practically zero, regardless of treatment. Biofilm formation has been one of the main virulence mechanisms of *Pseudomonas* hence presenting resistance to antibiotic therapy. We have manufactured chitosan nanoparticles (NQo) with tripolyphosphate (TPP) using ionotropic gelation. These NQo were confronted against a *Pseudomonas* sp. strain isolated from milk samples of cows diagnosed with BM, to evaluate their antimicrobial and antibiofilm capacity. The NQo showed great antibacterial effect in the minimum inhibitory concentrations (MIC), minimum bactericidal concentration (MBC) and disk diffusion assays. Using sub lethal concentrations, NQo were tested for inhibition of biofilm formation. The results show that the nanoparticles exhibited biofilm inhibition and were capable of eradicate pre-existing mature biofilm. These findings indicate that the NQo could act as a potential alternative to antibiotic treatment of BM.

## 1. Introduction

Bovine mastitis (BM) is an inflammation disease of the mammary gland caused by various pathogens and has disastrous consequences for the dairy industry [1]. It is one of the main infectious diseases of dairy cattle, becoming a global issue [2]. BM causes enormous economic losses due to poor milk quality, abuse of drugs and veterinary services, culminating in the elimination of the infected animals [3]. Misuse and overuse of antibiotics for the treatment and prevention of bovine mastitis leads to the development of resistance among mastitis pathogens [4]. Gram-negative bacteria play a role as causative pathogens in environmental-mastitis in the cattle [5,6] and these microorganisms represent 40% of all clinical mastitis cases (CM) [7]. Bacteria of the *Pseudomonas* genus has been identified as a predominant milk-associated psychotropic bacterium, becoming an important opportunistic pathogen in the dairy industry [8]. The intramammary infection by *Pseudomonas* sp. remains one of the most refractory to antibiotic therapy [9]. Improved dairy herd practices have remained largely ineffective at reducing the incidence of intramammary infections caused by Gram-negative bacteria [10]. The management of *Pseudomonas* infections in BM represents an increased challenge due to long persistence and spread capacity throughout a herd [11,12]. *Pseudomonas* sp. is widespread in the environment of dairy cows where the major sources in farms are multiple water supplies, soil, contaminated teat dips and infusion equipment [13]. The antibiotic recalcitrance associated with *Pseudomonas* can be mainly attributed to the formation of biofilms in different surfaces [14,15]. *Pseudomonas* is a versatile and adaptable pathogen that can cause infections of both medical and veterinary importance [7].

On the other hand, biofilms are a community conglomerate of microorganisms where cells adhere to each other embedded in a self-produced matrix composed of exopolysaccharides, extracellular DNA and proteins [16]. *Pseudomonas* biofilm formation and exopolysaccharide overproduction grants survival in adverse environmental conditions, contributing to pathogenesis, infections and resistance to conventional antibiotics [17,18]. Therefore, there is an urgent need of alternative treatments in farm animals to reduce antibiotic treatments [19]. Extensive research is required to find a new biocompatible and non-toxic antibiofilm molecule with a natural biological origin [20].

In this context, chitosan (Qo) is a molecule with a natural biological origin. Chitosan is a polysaccharide derived from chitin (the second most abundant polymer in nature) and is essentially composed of (β)-1,4 d-glucosamine linked to *N*-Acetyl-d-glucosamine residues [21,22]. Qo displays unique properties such as biodegradability and biocompatibility proving to be a simple and cost-effective alternative with many applications such as food safety and biomedicine [23,24]. Qo is known to have antibacterial and antibiofilm properties in Gram-positive bacteria such as *Staphylococcus aureus* and Gram-negative like *Escherichia coli* and *Salmonella typhimurium* [25,26,27].

However, Qo applications have faced difficulties due to their limited aqueous solubility at neutral pH and high viscosity [28,29]. A suitable strategy to facilitate the applications of Qo in biomedicine and pharmaceutical technology is the formulation of Qo nanoparticles (NQo), because the manufacture of nanoparticles from carbohydrate biopolymers improves the limiting parameters of the application [30]. Nanoparticles have recently attracted much attention as antimicrobial agents [31]. It has been reported that NQo have an antimicrobial activity in *Staphylococcus aureus* isolated from BM [32]. However, the antimicrobial activity of NQo against BM *Pseudomonas* pathogen has been poorly explored.

Therefore, all this background points Qo as a promising material to obtain nanoparticles to use as new therapeutic agents for BM treatment. The aim of this study is to evaluate and determine the antimicrobial and antibiofilm potential of NQo on a strain of *Pseudomonas* sp. isolated from milk samples from cows diagnosed with BM.

## 2. Results

### 2.1. Characterization of Chitosan Nanoparticles (NQo)

**Figure 1:**
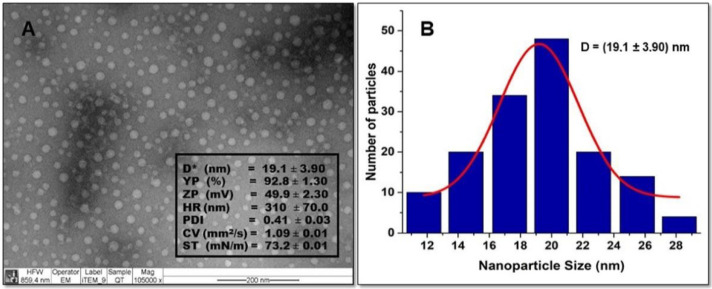
(**A**) Transmission electron microscopy (TEM) of NQo. Physicochemical characterization parameters: D*, diameter (nm); YP, yield particle (%); ZP, Zeta potential (mV); HR, hydrodynamic radius (nm); PDI, polydispersity index; CV, cinematic viscosity (mm^2^/s); ST, surface tension (mN/m). (**B**) Histogram showing the NQo size distribution.

The morphology of the particles was observed by TEM and is shown in Figure 1A. The TEM studies of the NQo were obtained against bright and dark fields. The micrographs show that NQo particles are spherical in shape with an average diameter of 19.1 ± 3.9 nm with a yield particle (%YP) of 92.8 ± 1.3%. The size of the particles is represented in a histogram shown in Figure 1B, to calculate the size distribution and normal deviation using the Image J 1.52v program.

The NQo prepared with the ionotropic gelation method have a Z-potential of 49.9 ± 2.3 mV, according to Caro et al. (2016) [33]. Figure 1 shows that the surface tension of 73.20 ± 0.01 (mN/m) and the kinematic viscosity value of the NQo is 1.09 ± 0.01 (mm^2^/s) in a temperature-controlled water bath (25 °C), both values are similar to those reported in Caro et al., 2016. The NQo scored a hydrodynamic diameter (Z-average = 310 ± 70 nm) that is different to the diameter measured by TEM (19.1 ± 3.9 nm). The polydispersity index (PDI) value was 0.4 ± 0.03. Finally, the stability of nanoparticles was determined by measuring Z-potential (+49.9 ± 2.3 mV).

### 2.2. Antimicrobial Capacity of NQo

#### 2.2.1. Minimum Inhibitory Concentration and Minimum Bactericidal Concentration

In order to test the NQo antimicrobial capacity, the nanoparticles were defied against the isolated *Pseudomonas* sp. strain. The minimum inhibitory concentration (MIC) and minimum bactericidal concentration (MBC) values of NQo against *Pseudomonas* sp. were found to be 280 μg/mL and 700 μg/mL, respectively (Table 1). This result was confirmed by absorbance measurements and viable cell count method which was quantified.

#### 2.2.2. Disk Diffusion Test and Comparison of NQo to Conventional Antibiotics

**Figure 2:**
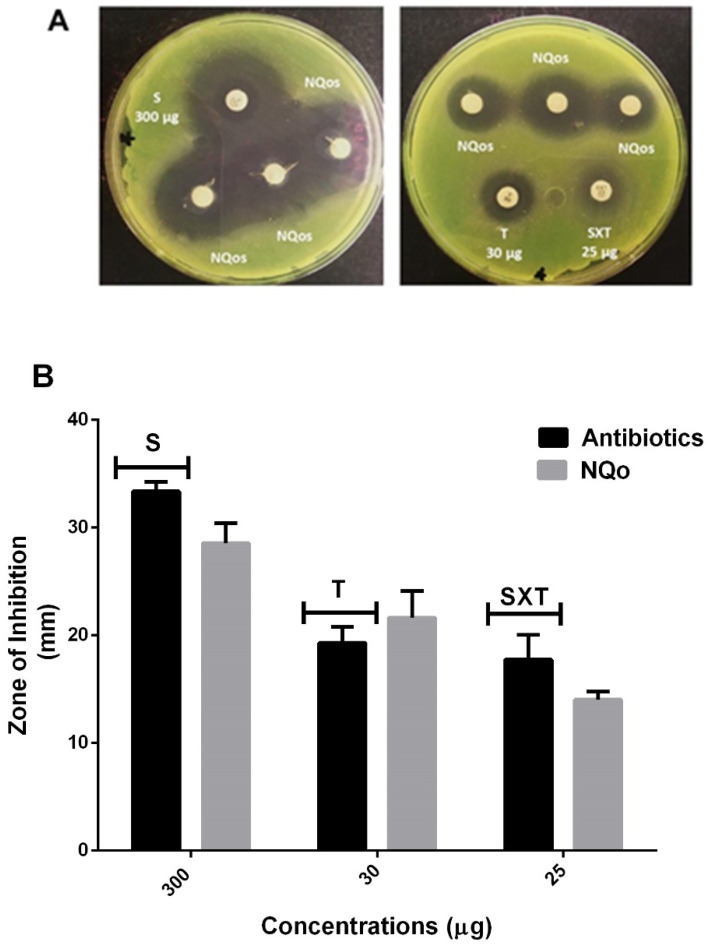
Growth inhibition zone of NQo on *Pseudomonas* sp. (**A**) Antibiogram photographs on agar; (**B**) graphical representation of comparative inhibition zone between conventional antibiotics. S = streptomycin; T = tetracycline; SXT = trimethoprim/sulfamethoxazole and NQo.

Another technique to evaluate the antimicrobial capacity of the NQo was the disk diffusion test (Kirky–Bauer method). Antimicrobial sensitivity of the isolated *Pseudomonas* sp. strain showed that it was sensitive to all the antibiotics tested. The results are shown in Figure 2A where the antibiotic streptomycin, at a concentration of 300 µg, showed a diameter of inhibition of 33.36 ± 0.89 mm in comparison to the disk loaded with NQo which, at the same concentration (300 µg), reached 28.55 ± 2.42 mm diameter of inhibition in average, hence the nanoparticles display almost the same inhibition zone. On the other hand, the antibiotic tetracycline at a concentration of 30 µg displayed an inhibition diameter of 19.97 ± 1.31 mm in comparison to the disk loaded with NQo that reached a 21.64 ± 2.21 mm, showing an ~8.35% larger inhibition zone than tetracycline. The antibiotic trimethoprim/sulfamethoxazole (SXT) at a concentration of 25 µg displayed an inhibition diameter of 17.1 ± 2.84 mm in comparison to the disk loaded with NQo at the same concentration that reached a 14.02 ± 0.47 mm, showing SXT to have a ~17.97% larger inhibition zone than NQo. These results show that NQo, in comparison to conventional antibiotics used in the treatment of *Pseudomonas* infections, are ~9.0% more effective at the same concentrations.

#### 2.2.3. Antibiofilm Capacity of NQo

**Figure 3:**
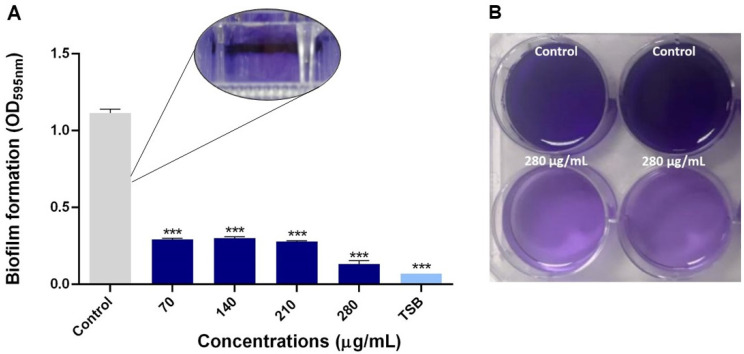
Effect of sub-MICs of NQo on *Pseudomonas* sp. biofilm formation at 37 °C: (**A**) Quantification of biofilm formation; (**B**) control and NQo MIC treatment (280 µg/mL) wells. *** *p* < 0.001 indicates statistical significance as compared to the control.

The NQo were examined for their biological activity against *Pseudomonas* sp. crystal violet staining assay has shown that in a concentration-dependent manner, inhibition of the biofilm formation was achieved by NQo at all tested sub-MICs concentrations. The results in Figure 3, show that at the lowest concentration tested (70 µg/mL) there was a 74.05 ± 0.83% biofilm inhibition compared to the control (without NQo treatment) and, at the MIC, the NQo shown an 88.96 ± 0.35% of biofilm formation inhibition within a 24 h treatment. These quantified results are shown in Table 2.

**Figure 4:**
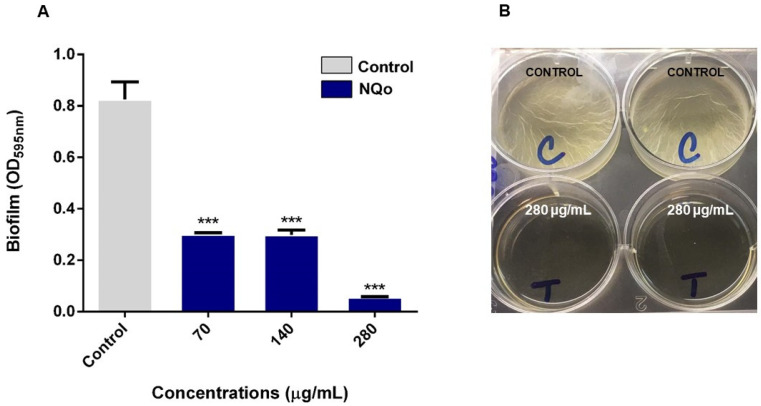
Effect of MIC NQo on *Pseudomonas* sp. biofilm formation at room temperature (25 °C): (**A**) quantification of biofilm formation; (**B**) control and NQo MIC treatment (280 µg/mL) wells. *** *p* < 0.001 indicates statistical significance as compared to the control.

On the other hand, results shown in Figure 4, correspond to the biofilm formation inhibition at room temperature (25 °C). It was found that, at this temperature, *Pseudomonas* forms a thicker and denser biofilm than at 37 °C and the NQo antibiofilm capacity intensifies, increasing the biofilm inhibition percentage up to 90%. As a comparison between the results obtained in the biofilm inhibition assays at 37 °C and room temperature (Figure 3 and Figure 4, respectively), against the ones at 25 °C shows that the biofilm formation phenotype intensifies so does the NQo antibiofilm effect.

**Figure 5:**
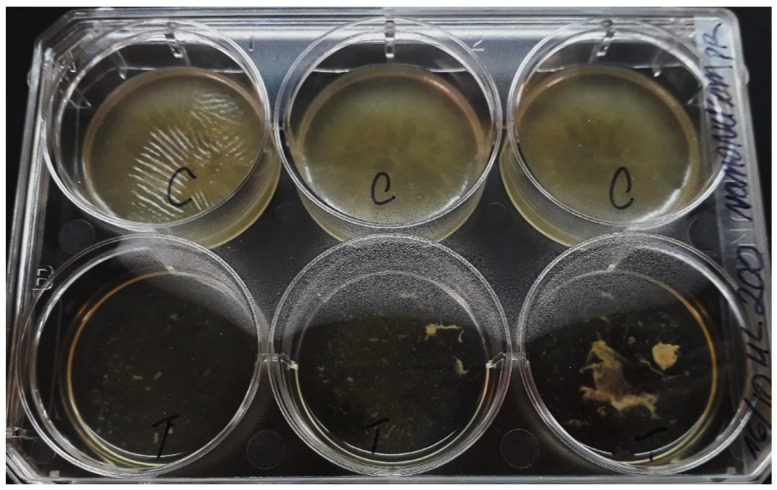
Biofilm eradication capacity and phenotype recovery of MIC NQo on *Pseudomonas* sp. at room temperature (25 °C).

When the biofilm eradication capacity and phenotype recovery of the MIC NQo were tested (Figure 5), the results showed a dramatic change in the phenotype that is likely irreversible. There was a mature biofilm eradication and subsequent loss of phenotype when tested for recovery. Although this result was not quantified by the crystal violet biofilm method, the change in the phenotype quality is evident. This result displays a significantly compromised structural integrity of the biofilms, overall loosening or achieving heterogeneous destruction of the biofilm matrix.

## 3. Discussion

### 3.1. Characterization of NQo

Table 1 shows the NQo physicochemical characterization. The NQo sizes, determined by TEM and the dynamic light scattering (DLS), are shown. Values determined using DSL were higher than those measured by TEM because the latter directly measures a dry sample, while the Zetasizer system determines the size by first measuring the random motion of particles in a liquid, due to the bombardment of the surrounding particles (Brownian motion) in a sample by DLS, and then extrapolating the size of this motion using the Stokes–Einstein equation [34,35].

The factors involved in the NQo size, such as the Qo used and the appropriate concentrations, decrease the probability of particle aggregation, producing a high Z-potential that indicates an incipient stability of the particles due to a strong electrostatic interaction, which causes no aggregation between them. This agrees with our results in which this relationship was observed by microscopy for NQo. According to Medina et al., 2019, the most important properties that affect antimicrobial activity are nanoparticle size and Z-potential [36,37,38,39,40]. Viscosity is a very important factor in this research since, when carried out at room temperature, it favors the formulation of an adequate viscosity to produce a direct diffusion and a good interaction of the Qo with *Pseudomonas* contact area (Gram-negative bacteria). Solving Qo applications has faced difficulties due to its limited aqueous solubility at neutral pH and high viscosity. The value indicated in Table 1 is adequate compared with other formulation prototypes to study Qo antimicrobial activity [41,42,43].

### 3.2. Antimicrobial Capacity of NQo

The minimum inhibitory concentration (MIC) and minimum bactericidal concentration (MBC) values of NQo against *Pseudomonas* sp. were found to be 210 μg/mL and 700 μg/mL, respectively (Table 2). The establishment of MIC and MBC are essential assays to determine the antimicrobial capacity of NQo against *Pseudomonas* sp., so it’s with that objective that a wide range of concentrations were tested. The literature indicates that the NQo MICs and the Qo itself against *Pseudomonas aeruginosa* vary from 128 to 4096 µg/mL [44], indicating that the results obtained in this research are favorable due to the range in which they are. Most importantly, in BM related pathogens, NQo MIC values ranged from 200 to 400 μg/mL for *S. aureus* strains isolated from BM [45]. The results shown in Figure 3, indicate that NQo, in comparison to conventional antibiotics used in the treatment of *Pseudomonas* infections, are equally or around 15% more effective at its antimicrobial effect. According to the CLSI, in order to show susceptibility and antimicrobial capacity must display a zone of inhibition (ZOI) above 11 nm, depending on the agent and its concentration [46]. Therefore, the results shown above indicate that the NQo have a great antimicrobial capacity. This difference might rely on the action mechanisms described for each antibiotic and for Qo. As far as the antibiotics tested, streptomycin is a bactericidal antibiotic that acts by binding to the 30S ribosomal subunit of susceptible organisms and disrupting the initiation and elongation steps in protein synthesis [47]. On the other hand, tetracycline is a broad-spectrum polyketide antibiotic that also binds specifically to the 30S ribosome of the bacteria, preventing the attachment of aminoacyl tRNA to the RNA-ribosome complex affecting the protein synthesis as well [48]. Finally, SXT blocks two steps in the bacterial biosynthesis of essential nucleic acids and proteins, thus having a bactericidal effect [49]. The mechanism of NQo antimicrobial capacity have been linked to the interactions and changes in the permeability and damages of the cell membrane, leading to modifications in the cell morphology and liberation of proteins and DNA which culminates in the death of the cell [50,51]. In the literature it has also been mentioned and demonstrated that Qo antibacterial properties depend on the molecular weight, the degree of deacetylation, the temperature plus the strain against which it is tested, the growth phase and the initial cell concentration [52]. The high surface-to-volume ratio of nanosized materials allows a different interaction behavior with bacteria due the increased local charge density, explaining with this the enhanced activity of NQo [53,54]. The mechanism behind its microbial activity is not very clear; however, loss of the cell wall integrity and consequent alteration in membrane permeability has been reported [48].

### 3.3. Biofilm Inhibition Properties of NQo

According to the results in Table 2 and Figure 4 and Figure 5, it has been reported that the mechanism of the chitosan antibiofilm property is mainly attributed to its polycationic nature given by the functional amino groups (NH_3_^+^) of *N*-acetylglucosamine units. The positive charge of Qo is expected to react electrostatically with the negatively charged biofilm components such as EPS, proteins and DNA, resulting in an inhibitory effect on bacterial biofilm [20].

At a cell membrane level, an electrostatically interaction occurs between the amine and phospholipids, causing a rise of the membrane permeability and inducing leakage of cytosolic components [51,55]. While the biofilm formation phenotype at 25 °C (room temperature) intensifies, so does the NQo antibiofilm effect. It has been stated in the literature that temperature, nutrient availability, oxygen, among other factors, affect the biofilm formation which represents a protective mode of growth that allows microorganisms to survive in hostile environments [56].

Antibacterial activity of Qo varies with molecular weight, charge and organism. Mellegard et.al., 2011, stated that Qo molecular weight and degree of deacetylation and derivates are important factors for their biological activities [57]. In a nanoparticle form, this polysaccharide can easily penetrate and eradicate the biofilm matrix while reducing its toxicity and working effectively at low concentrations [58].

Although *Pseudomonas* is known for its intrinsic resistance to antibiotics due to the production of enzymes, upregulation of efflux pumps and its ability to acquire external genes encoding resistance to a variety of antimicrobial agents [59,60,61].

### 3.4. Eradication of NQo to Established Mature Pseudomonas sp. Biofilm and Phenotype Recovery

Dispersion of the mature biofilm of *Pseudomonas* sp. is also equally important as inhibiting its formation. In fact, it responds to a completely different phenotype and to a different problem as well, because the first situation obeys to an avoidance of the formation and the other is a rescue or disruption of the established biofilm state, which could be translated to an established *Pseudomonas* infection in a mammalian organ or surface [62]. Hence, it is fundamental to evaluate the NQo efficacy in eradicating the mature *Pseudomonas* biofilm. In the present study, Figure 5 results show that NQo presence at MIC significantly dispersed the stablished (96 h) mature biofilm of *Pseudomonas* sp. as compared to the control. The eradicating property of chitosan oligosaccharides (COS) and derivates has been reported in the literature [21,63]. Additionally, the results show a dramatic change in the phenotype. This result displays a significantly compromised structural integrity of the biofilms, overall loosening of the biofilm or heterogeneous destruction of the biofilm matrix. It has been stated in the literature that Qo can interact with DNA in an electrostatic interaction, the polymer has a polycationic nature that interacts with the negative charge of the DNA, being essential to the formation of *Pseudomonas* sp. biofilm [64]. Indeed, cleavage of DNA by DNase decreases the structural integrity of such biofilms [65]. Additionally, there is a chance of interaction with other biofilm matrix components that influence greatly to the structural integrity of it, reducing more than just eDNA biofilm viscoelasticity and even interfering with the Quorum Sensing pathways [66,67]. As the *Pseudomonas* biofilm matrix is comprised predominantly of anionic macromolecules such as exopolysaccharides [68], the introduction of cationic chitosan alters the biofilm electrostatics and potentially enhances the antibacterial activities of NQo. Studies have shown that the exopolysaccharides provide strength, cohesion and the capacity to retain large and small molecules alike [69]. The biofilm formation is a subsequently concerted process beginning with the attachment to an abiotic or biotic surface that culminates in the formation and maturation of the structured system that presents nutrients and oxygen gradients, setting up a microenvironment with a series of ecological relations related to the occupation of a niche, securing the success of the biofilm [16,70]. Finally, according to the literature, it has been stated the phenotype loss after the dispersal with antimicrobial agents, when concentration is above the saturation point of diffusion/reaction inhibition [56]. A better understanding of the genetics and molecular mechanisms of biofilm formation may provide some insights for the control of problems related to biofilm formation.

## 4. Materials and Methods

### 4.1. Bacterial Strain, Isolation and Culture Conditions

The strain of *Pseudomonas* used in this study was isolated from milk samples obtained from symptomatic Holstein Fresian cows that were diagnosed to have clinical mastitis by veterinarians. These cows were from Los Muermos, X Región de Los Lagos, Chile. The specimens presented inflammation of the mammary gland, fibrosis of the udder, high count of somatic cells, scored grade 3 in the California Mastitis Test, and were without prior treatment when the milk samples were obtained. The isolation of *Pseudomonas* strain was carried out according to the recommendations of the International Dairy Federation [71].

For colonies isolation of *Pseudomonas* sp., typical colonies were selected, specifically those that showed a spontaneous pale green and fluorescent green color in ultraviolet light on Cetrimide agar, oxidase positive [72]. After phenotypic identification on agar and biochemical tests, DNA was extracted from characteristic colonies on Cetrimide agar using the Wizard^®^ Genomic DNA Purification Kit (Promega). The purity of the extracted bacterial DNA was assessed using the Nanodrop ND-1000 and the quantity of bacterial DNA was measured using the Qubit1 2.0 Fluorometer and were amplified by PCR using the partial sequence of the 16S gene (rRNA) (F: 5′-GAGTTTGATCCTGGCTCAG-3′ and R: 5′–ATTACCGCGGCTGCTGG-3′), which was sequenced by Macrogen (https://dna.macrogen.com/). The received sequence was analyzed by alignment BLAST (https://blast.ncbi.nlm.nih.gov/Blast.cgi) in which identification of the species was obtained, according to sequence comparison, when the greatest similarity with the identity of the bacteria proposed in the program was found (https://www.ncbi.nlm.nih.gov/nucleotide/674119453?report=genbank&log$=nuclalign&blast_rank=1&RID=HKW5ZNR1015) [73].

After phenotypic and genotypic characterization of *Pseudomonas* sp., the strain was cultivated and tested in Tryptic Soy Broth (TSB) for MIC and MBC antimicrobial testing, Tryptic Soy Agar (TSA) for colony forming unit recount (CFU) and Mueller Hinton Agar (MH) for disk diffusion test culture media plates according to Khan et al., 2019. The temperature for bacterial culture was 37 °C, except for the Biofilm formation protocol at room temperature (25 °C). Low molecular weight Chitosan (269 kDa) was purchased from Sigma-Aldrich, Inc. (St. Louis, MO, USA, C448869). The acetylation degree was 88.5% measured by proton nuclear magnetic resonance [36].

### 4.2. Synthesis of Chitosan Nanoparticles (NQo)

Chitosan nanoparticles (NQo) were synthesized using ionotropic gelation according to Caro et al., 2016 and Medina et al., 2019 [33,36]. Briefly, 0.3% (*w/v*) of low-molecular weight Qo was prepared in 0.1 M acetic acid and stirred for 24 h. The Qo solution was mixed in 2:1 (*v/v*) ratio with a solution of tripolyphospate (TPP) 0.1% (*w/v*) and added dropwise (infusion pump KDS200, KD Scientific©) (1.8 mL/min) with constant magnetic stirring at room temperature. The suspension obtained was centrifuged at 21,000× *g* for 30 min at 14 °C (HermLe model Z32K, Wehingen, Germany).

### 4.3. Characterization of NQo

#### 4.3.1. Cinematic Viscosity Determination

Absolute viscosity dispersions of NQo were measured using an Ostwald U-tube viscometer (size B, Technico, UK) in a temperature-controlled water bath (25 °C) (Grant Instruments Ltd., Cambridge, UK). Cinematic viscosity was calculated using Stokes formula and expressed in mm^2^/s [33].

#### 4.3.2. Surface Tension Measurements

Surface tension was measured with a Kibron microtensiometer (Kibron Inc., Helsinki, Finland) using 50 mL of the NQo dispersion sample. The instrument was calibrated with distilled water as a reference (surface tension = 72.8 mN m^−1^ at 25 °C) [33].

#### 4.3.3. Determination of Size, Z Potential and Polydispersity Index

The variation in size and surface load was determined, this measurement was performed using the Zetasizer Nano ZS-90 equipment (Malvern Instruments). 1.0 mL of the NQo suspension was taken and deposited in a folded polystyrene capillary cuvette (model s90). The analyses with the equipment were carried out under standard conditions (dispersant: water, T: 25 °C, laser: 633 nm)

#### 4.3.4. Transmission Electron Microscopy (TEM)

For the size determinations of the NQo, besides the Zetasizer measurements, a Philips Tecnai 12 Bio Twin transmission electron microscope was used. One drop of nanoparticles was spread onto a coated copper grid (SPI Supplies, Inc., West Chester, PA, USA) that was dried before TEM analysis.

### 4.4. Determination of Minimum Inhibitory Concentration (MIC) and Minimum Bactericidal Concentrations (MBC) of NQo

The MIC and MBC of NQo against *Pseudomonas* was determined by following the guidelines of the Clinical and Laboratory Standards Institute [74]. An overnight grown bacterial culture of *Pseudomonas* sp. was diluted at a turbidity of 0.05 at 600 nm optical density (OD_600_) and were used in the presence and absence of NQo. A volume of 400 μL of diluted cell culture was transferred to different assay tubes and treated with NQo at concentrations that ranged from 14 to 1400 μg/mL, in triplicates. The compound and culture containing were incubated at 37 °C for 24 h in shaking conditions. After incubation, the OD_600_ of the culture was measured using the Infinite m200 Pro TECAN Plate Reader. The MIC that was recorded was the lowest concentration that resulted in no visible growth of microorganisms after 24 h of incubation at 37 °C (no turbidity). The same treatment conditions were used to determine MBC by using the plate count of viable cells method (CFU). Briefly, a volume of 1000 μL of culture exposed to NQo was inoculated in 9.0 mL of physiological serum (NaCl 0.9% *w/v*) and serial dilutions were made up to 10^−5^. The mixed cell suspension (1000 μL) was spread plated onto a TSA agar plate and incubated at 37 °C for 24 h. After incubation, the colonies that appeared on the TSA agar plate were counted. Where the result was expressed as cfu/mL. The lowest concentration capable of killing 99.9% of the starting inoculum was defined as the MBC [75].

### 4.5. Disk Diffusion Test and Comparison of NQo to Conventional Antibiotics

The agar diffusion method was used to determine the antibacterial performance of the NQo compared to conventional antibiotics and its efficiency against *Pseudomonas* isolated from MB, the assays were carried out according to the methodology recommended by Bauer, Kirby, Sherris, and Turck (1966) and the NCCLS (National Committee for Clinical Laboratory Standards, 1990) [73,76].

The Mueller–Hinton agar plates were inoculated with bacterial suspensions (100 µL of ~1 × 10^6^ CFU/mL). Blank disks were loaded with NQo equivalent to antibiotic concentrations to compare: Streptomycin (300 µg), gentamicin (10 µg), tetracycline (30 µg), and sulfamethoxazole/trimethoprim (25 µg). The plates were incubated at 37 °C for 24 h, and the inhibition zones were estimated by measuring the diameters of the areas with no microorganism growth and were compared with the zones of inhibition of conventional antibiotics. This experiment was performed in triplicates.

### 4.6. Evaluation of Biofilm Inhibition and Eradication Properties of NQo

#### 4.6.1. Biofilm Inhibition by NQo

The antibiofilm efficacy of NQo at sub-MICs was quantified by using crystal violet method [25]. For this assay, 2 strategies were used. The first one was at 37 °C using a 96-well polystyrene microtiter plate that was used as the surface for the bacterial biofilm formation. The *Pseudomonas* sp. cell culture that grown overnight were diluted at a turbidity of 0.05 (OD_600_) and were used for biofilm formation in the presence and absence of NQo. Various sub-MICs were added to the 96-well microtiter plate containing 250 μL of cell culture and were incubated at 37 °C for 24 h. The free-floating (planktonic) cells were discarded after incubation and methanol fixation, followed by staining with crystal violet. The biofilm cells were washed three times with water and re-suspended in 33% (*v/v*) glacial acetic acid and were measured at OD_570_ by an Infinite m200 Pro TECAN Plate Reader. The second strategy, similar to the first, but using room temperature instead, tried to mimic environmental conditions. A 6-well polystyrene plate was used as a surface for the bacterial biofilm formation containing 400 μL of cell culture and was incubated at 25 °C for 48 h.

#### 4.6.2. Mature Biofilm Eradication and Recovery of the Phenotype

To test the biofilm eradication capacity and recovery of the phenotype using the NQo, the biofilm formation protocol was used at room temperature, where in a 6-well microtiter plate 400 µL of cell culture diluted was added to fill with TSB media, then it was incubated at room temperature (25 °C) for 96 h, then at day five, the MIC of NQo was added to the wells, incubating it at 25 °C for 24 h more. Next day, the free-floating (planktonic) cells were discarded after incubation. Finally, an equal volume of TSB was added to the 6-well plates and it was incubated for 24 h at room temperature.

### 4.7. Statistical Analysis

All experiments were carried out in triplicates, the graphs presented in this study were constructed by GraphPad Prism 6.0 (GraphPad Software Inc., San Diego, CA, USA) and the results were presented as means ± standard deviation (SD). The statistical analysis of each data was performed using one-way ANOVA followed by Tukey a posteriori test. Differences with a *p* < 0.001 were considered statistically significant.

## 5. Conclusions

In this study, an ionic gelation method was developed to generate stable NQo. These NQo were tested against a strain of *Pseudomonas* sp. isolated from BM infection although does not represents to all *Pseudomonas* around the world, it is an initial analysis that allows us to see the effectiveness of these nanoparticles against wild strains isolated from infection. The activity included antimicrobial testing, inhibition of biofilm at the initial stage and dispersion of the stablished mature biofilm. The NQo had an excellent antimicrobial capacity and were able to effectively eradicate preformed biofilm and inhibit its formation. These findings indicate that the NQo formulation could act as a potential alternative to antibiotic treatment of BM and could be applied as a dipping solution for the udder and/or generate a prototype intramammary pommel for the control or treatment of BM which will involve additional studies to our results, such as physicochemical analysis of the stability formulation and pharmacokinetic analysis. Although, this study does not elucidate a possible mechanism, the study of changes in gene expression in bacteria under NQo treatment could be an indicator factor to evaluate the precise antibacterial activities of NQo at a genotypical level, this way a more specific molecular mechanism would be described. Polymeric Np have not yet been deeply explored in BM therapy, even when they can act as antimicrobial agents and nanocarriers at the same time, thus allowing the design of powerful combined therapies.

Subsequent studies could confirm the antimicrobial and antibiotic properties against the phenotype also at the genotypic level, studying biofilm formation, exopolysaccharide synthesis, virulence factor-producing genes and quorum sensors. Additionally, cytotoxicity in bovine cell lines. For this reason, it is relevant to evaluate the use of alternative antimicrobial molecules for control and treatment of bovine mastitis.

## Figures and Tables

**Table 1:** Susceptibility of *Pseudomonas* sp. to NQo.

MIC NQo (µg/mL)	MBC NQo (µg/mL)	MBC/MIC
280	700	2.5

**Table 2:** Biofilm inhibition of *Pseudomonas* sp. treated with NQo.

NQo (µg/mL)	Biofilm Inhibition (%)
0	0
70	74.05 ± 0.83
140	73.28 ± 0.84
210	75.10 ± 0.65
280	88.96 ± 0.35

## References

[1] Martins S.A.M., Martins V.C., Cardoso F.A., Germano J., Rodrigues M., Duarte C., Bexiga R., Cardoso S., Freitas P.P. (2019). Biosensors for on-farm diagnosis of mastitis. Front. Bioeng. Biotechnol..

[2] Ruegg P.L. (2017). A 100-Year Review: Mastitis detection, management, and prevention. J. Dairy Sci..

[3] Aslantaş Ö., Demir C. (2016). Investigation of the antibiotic resistance and biofilm-forming ability of Staphylococcus aureus from subclinical bovine mastitis cases. J. Dairy Sci..

[4] Fessia A.S., Dieser S.A., Raspanti C.G., Odierno L.M. (2019). Genotyping and study of adherence-related genes of Streptococcus uberis isolates from bovine mastitis. Microb. Pathog..

[5] Angelopoulou A., Holohan R., Rea M.C., Warda A.K., Hill C., Ross R.P. (2015). Bovine mastitis is a polymicrobial disease requiring a polydiagnostic approach. Int. Dairy J..

[6] Klaas I.C., Zadoks R.N. (2018). An update on environmental mastitis: Challenging perceptions. Transbound. Emerg. Dis..

[7] Schukken Y., Chuff M., Moroni P., Gurjar A., Santisteban C., Welcome F., Zadoks R. (2012). The “Other” Gram-Negative Bacteria in Mastitis. Klebsiella, Serratia, and More. Vet. Clin. N. Am. Food Anim. Pract..

[8] Meng L., Zhang Y., Liu H., Zhao S., Wang J., Zheng N. (2017). Characterization of *Pseudomonas* spp. and associated proteolytic properties in raw milk stored at low temperatures. Front. Microbiol..

[9] Park H., Hong M., Hwang S., Park Y., Kwon K., Yoon J., Shin S., Kim J., Park Y. (2014). Characterisation of *Pseudomonas aeruginosa* related to bovine mastitis. Acta Vet. Hung..

[10] Bannerman D.D., Chockalingam A., Paape M.J., Hope J.C. (2005). The bovine innate immune response during experimentally-induced *Pseudomonas aeruginosa* mastitis. Vet. Immunol. Immunopathol..

[11] Kelly E.J., Wilson D.J. (2016). *Pseudomonas aeruginosa* mastitis in two goats associated with an essential oil–based teat dip. J. Vet. Diagn. Investig..

[12] McDougall S., Arthur D.G., Bryan M.A., Vermunt J.J., Weir A.M. (2007). Clinical and bacteriological response to treatment of clinical mastitis with one of three intramammary antibiotics. N. Z. Vet. J..

[13] Vidal A.M.C., Netto A.S., Vaz A.C.N., Capodifóglio E., Gonçalves A.C.S., Rossi G.A.M., Figueiredo A.S., Ruiz V.L.A. (2017). *Pseudomonas* spp.: Contamination sources in bulk tanks of dairy farms. Pesqui. Vet. Bras..

[14] Bassetti M., Vena A., Croxatto A., Righi E., Guery B. (2018). How to manage *Pseudomonas aeruginosa* infections. Drugs Context.

[15] Pang Z., Raudonis R., Glick B.R., Lin T.J., Cheng Z. (2019). Antibiotic resistance in *Pseudomonas aeruginosa*: Mechanisms and alternative therapeutic strategies. Biotechnol. Adv..

[16] Flemming H.C., Neu T.R., Wozniak D.J. (2007). The EPS matrix: The “House of Biofilm Cells”. J. Bacteriol..

[17] Moradali M.F., Ghods S., Rehm B.H.A. (2017). *Pseudomonas aeruginosa* lifestyle: A paradigm for adaptation, survival, and persistence. Front. Cell. Infect. Microbiol..

[18] Kirchhelle C. (2018). Pharming animals: A global history of antibiotics in food production (1935–2017). Palgrave Commun..

[19] Roy R., Tiwari M., Donelli G., Tiwari V. (2018). Strategies for combating bacterial biofilms: A focus on anti-biofilm agents and their mechanisms of action. Virulence.

[20] Khan F., Pham D.T.N., Oloketuyi S.F., Manivasagan P., Oh J., Kim Y.M. (2020). Chitosan and their derivatives: Antibiofilm drugs against pathogenic bacteria. Colloids Surf. B Biointerfaces.

[21] Liaqat F., Eltem R. (2018). Chitooligosaccharides and their biological activities: A comprehensive review. Carbohydr. Polym..

[22] Youssef A.M., Abou-Yousef H., El-Sayed S.M., Kamel S. (2015). Mechanical and antibacterial properties of novel high performance chitosan/nanocomposite films. Int. J. Biol. Macromol..

[23] Najafabadi S.A.A., Mohammadi A., Kharazi A.Z. (2020). Polyurethane nanocomposite impregnated with chitosan-modified graphene oxide as a potential antibacterial wound dressing. Mater. Sci. Eng. C.

[24] Xie M., Huang K., Yang F., Wang R., Han L., Yu H., Ye Z., Wu F. (2020). Chitosan nanocomposite films based on halloysite nanotubes modification for potential biomedical applications. Int. J. Biol. Macromol..

[25] Khan F., Manivasagan P., Lee J.W., Pham D.T.N., Oh J., Kim Y.M. (2019). Fucoidan-stabilized gold nanoparticle-mediated biofilm inhibition, attenuation of virulence and motility properties in *Pseudomonas aeruginosa* PAO1. Mar. Drugs.

[26] Paomephan P., Assavanig A., Chaturongakul S., Cady N.C., Bergkvist M., Niamsiri N. (2018). Insight into the antibacterial property of chitosan nanoparticles against Escherichia coli and Salmonella Typhimurium and their application as vegetable wash disinfectant. Food Control.

[27] Huang J., Liu Y., Yang L., Zhou F. (2019). Synthesis of sulfonated chitosan and its antibiofilm formation activity against E. coli and S. aureus. Int. J. Biol. Macromol..

[28] Shah A., Ashames A.A., Buabeid M.A., Murtaza G. (2020). Synthesis, in vitro characterization and antibacterial efficacy of moxifloxacin-loaded chitosan-pullulan-silver-nanocomposite films. J. Drug Deliv. Sci. Technol..

[29] Muslim S.N., Kadmy I.M.S.A., Ali A.N.M., Salman B.K., Ahmad M., Khazaal S.S., Hussein N.H., Muslim S.N. (2018). Chitosan extracted from Aspergillus flavus shows synergistic effect, eases quorum sensing mediated virulence factors and biofilm against nosocomial pathogen *Pseudomonas aeruginosa*. Int. J. Biol. Macromol..

[30] Kaur P., Choudhary A., Thakur R. (2013). Synthesis of chitosan-silver nanocomposites and their antibacterial activity. Int. J. Sci. Eng. Res..

[31] Wang W., Arshad M.I., Khurshid M., Rasool M.H., Nisar M.A., Aslam M.A., Qamar M.U. (2018). Antibiotic resistance: A rundown of a global crisis. Infect. Drug Resist..

[32] Orellano M.S., Isaac P., Breser M.L., Bohl L.P., Conesa A., Falcone R.D., Porporatto C. (2019). Chitosan nanoparticles enhance the antibacterial activity of the native polymer against bovine mastitis pathogens. Carbohydr. Polym..

[33] Caro N., Medina E., Díaz-Dosque M., López L., Abugoch L., Tapia C. (2016). Novel active packaging based on films of chitosan and chitosan/quinoa protein printed with chitosan-tripolyphosphate-thymol nanoparticles via thermal ink-jet printing. Food Hydrocoll..

[34] Yue Y., Kan Y., Choi H., Clearfield A., Liang H. (2015). Correlating hydrodynamic radii with that of two dimensional nanoparticles. Appl. Phys. Lett..

[35] Stetefeld J., McKenna S.A., Patel T.R. (2016). Dynamic light scattering: A practical guide and applications in biomedical sciences. Biophys. Rev..

[36] Medina E., Caro N., Abugoch L., Gamboa A., Díaz-Dosque M., Tapia C. (2019). Chitosan thymol nanoparticles improve the antimicrobial effect and the water vapour barrier of chitosan-quinoa protein films. J. Food Eng..

[37] Antoniou J., Liu F., Majeed H., Qi J., Yokoyama W., Zhong F. (2015). Physicochemical and morphological properties of size-controlled chitosan-tripolyphosphate nanoparticles. Colloids Surf. A Physicochem. Eng. Asp..

[38] Shaban S.M., Aiad I., El-Sukkary M.M., Soliman E.A., El-Awady M.Y. (2017). Synthesis of newly cationic surfactant based on dimethylaminopropyl amine and their silver nanoparticles: Characterization; surface activity and biological activity. Chin. Chem. Lett..

[39] Li X., Wang R., Shi H., Song B. (2018). Effective surface tension of liquid marbles using controllable nanoparticle monolayers. Appl. Phys. Lett..

[40] Shaban S.M., Abd-Elaal A.A. (2017). Studying the silver nanoparticles influence on thermodynamic behavior and antimicrobial activities of novel amide Gemini cationic surfactants. Mater. Sci. Eng. C.

[41] Kim K.W., Min B.J., Kim Y.T., Kimmel R.M., Cooksey K., Park S.I. (2011). Antimicrobial activity against foodborne pathogens of chitosan biopolymer films of different molecular weights. LWT Food Sci. Technol..

[42] Divya K., Vijayan S., George T.K., Jisha M.S. (2017). Antimicrobial properties of chitosan nanoparticles: Mode of action and factors affecting activity. Fibers Polym..

[43] Wang L., Hu C., Shao L. (2017). The-antimicrobial-activity-of-nanoparticles--present-situation. Int. J. Nanomed..

[44] Qi L., Xu Z., Jiang X., Hu C., Zou X. (2004). Preparation and antibacterial activity of chitosan nanoparticles. Carbohydr. Res..

[45] Felipe V., Breser M.L., Bohl L.P., Rodrigues da Silva E., Morgante C.A., Correa S.G., Porporatto C. (2019). Chitosan disrupts biofilm formation and promotes biofilm eradication in Staphylococcus species isolated from bovine mastitis. Int. J. Biol. Macromol..

[46] CLSI (2017). Performance Standards for Antimicrobial Susceptibility Testing.

[47] Demirci H., Murphy F., Murphy E., Gregory S.T., Dahlberg A.E., Jogl G. (2013). A structural basis for streptomycin-induced misreading of the genetic code. Nat. Commun..

[48] Nguyen F., Starosta A.L., Arenz S., Sohmen D., Dönhöfer A., Wilson D.N. (2014). Tetracycline antibiotics and resistance mechanisms. Biol. Chem..

[49] Wei C., Ni W., Cai X., Zhao J., Cui J. (2016). Evaluation of trimethoprim/sulfamethoxazole (SXT), minocycline, tigecycline, moxifloxacin, and ceftazidime alone and in combinations for sxt-susceptible and sxt-resistant stenotrophomonas maltophilia by in vitro time-kill experiments. PLoS ONE.

[50] Helander I.M., Nurmiaho-Lassila E.L., Ahvenainen R., Rhoades J., Roller S. (2001). Chitosan disrupts the barrier properties of the outer membrane of Gram-negative bacteria. Int. J. Food Microbiol..

[51] Liu H., Du Y., Wang X., Sun L. (2004). Chitosan kills bacteria through cell membrane damage. Int. J. Food Microbiol..

[52] Foster L.J.R., Ho S., Hook J., Basuki M., Marçal H. (2015). Chitosan as a biomaterial: Influence of degree of deacetylation on its physiochemical, material and biological properties. PLoS ONE.

[53] Shrestha A., Zhilong S., Gee N.K., Kishen A. (2010). Nanoparticulates for antibiofilm treatment and effect of aging on its antibacterial activity. J. Endod..

[54] Joo S.H., Aggarwal S. (2018). Factors impacting the interactions of engineered nanoparticles with bacterial cells and biofilms: Mechanistic insights and state of knowledge. J. Environ. Manag..

[55] Khan F., Lee J.W., Manivasagan P., Pham D.T.N., Oh J., Kim Y.M. (2019). Synthesis and characterization of chitosan oligosaccharide-capped gold nanoparticles as an effective antibiofilm drug against the *Pseudomonas aeruginosa* PAO1. Microb. Pathog..

[56] Flemming H.C., Wingender J., Szewzyk U., Steinberg P., Rice S.A., Kjelleberg S. (2016). Biofilms: An emergent form of bacterial life. Nat. Rev. Microbiol..

[57] Mellegard H., Strand S.P., Christensen B.E., Granum P.E., Hardy S.P. (2011). Antibacterial activity of chemically defined chitosans: Influence of molecular weight, degree of acetylation and test organism. Int. J. Food Microbiol..

[58] García-Lara B., Saucedo-Mora M.A., Roldán-Sánchez J.A., Pérez-Eretza B., Ramasamy M., Lee J., Coria-Jimenez R., Tapia M., Varela-Guerrero V., García-Contreras R. (2015). Inhibition of quorum-sensing-dependent virulence factors and biofilm formation of clinical and environmental *Pseudomonas aeruginosa* strains by ZnO nanoparticles. Lett. Appl. Microbiol..

[59] Franklin M.J., Nivens D.E., Weadge J.T., Lynne Howell P. (2011). Biosynthesis of the *Pseudomonas aeruginosa* extracellular polysaccharides, alginate, Pel, and Psl. Front. Microbiol..

[60] Wei Q., Ma L.Z. (2013). Biofilm matrix and its regulation in *Pseudomonas aeruginosa*. Int. J. Mol. Sci..

[61] Ayoub Moubareck C., Hammoudi Halat D., Akkawi C., Nabi A., AlSharhan M.A., AlDeesi Z.O., Peters C.C., Celiloglu H., Karam Sarkis D. (2019). Role of outer membrane permeability, efflux mechanism, and carbapenemases in carbapenem-nonsusceptible *Pseudomonas aeruginosa* from Dubai hospitals: Results of the first cross-sectional survey. Int. J. Infect. Dis..

[62] Da Costa Lima J.L., Alves L.R., Da Paz J.N.P., Rabelo M.A., Maciel M.A.V., De Morais M.M.C. (2017). Analysis of biofilm production by clinical isolates of *Pseudomonas aeruginosa* from patients with ventilator-Associated pneumonia. Rev. Bras. Ter. Intensiva.

[63] Jiang F., Deng Y., Yeh C.K., Sun Y. (2014). Quaternized chitosans bind onto preexisting biofilms and eradicate pre-attached microorganisms. J. Mater. Chem. B.

[64] Orgaz B., Lobete M.M., Puga C.H., Jose C.S. (2011). Effectiveness of chitosan against mature biofilms formed by food related bacteria. Int. J. Mol. Sci..

[65] Reighard K.P., Hill D.B., Dixon G.A., Worley B.V., Schoenfisch M.H. (2015). Disruption and eradication of *P. aeruginosa* biofilms using nitric oxide-releasing chitosan oligosaccharides. Biofouling.

[66] Brindhadevi K., LewisOscar F., Mylonakis E., Shanmugam S., Verma T.N., Pugazhendhi A. (2020). Biofilm and Quorum sensing mediated pathogenicity in *Pseudomonas aeruginosa*. Process Biochem..

[67] Badawy M.S.E.M., Riad O.K.M., Taher F.A., Zaki S.A. (2020). Chitosan and chitosan-zinc oxide nanocomposite inhibit expression of LasI and RhlI genes and quorum sensing dependent virulence factors of *Pseudomonas aeruginosa*. Int. J. Biol. Macromol..

[68] Zhang T., Pabst B., Klapper I., Stewart P.S. (2013). General theory for integrated analysis of growth, gene, and protein expression in biofilms. PLoS ONE.

[69] Cornforth D.M., Dees J.L., Ibberson C.B., Huse H.K., Mathiesen I.H., Kirketerp-Møller K., Wolcott R.D., Rumbaugh K.P., Bjarnsholt T., Whiteley M. (2018). *Pseudomonas aeruginosa* transcriptome during human infection. Proc. Natl. Acad. Sci. USA.

[70] Han C., Goodwine J., Romero N., Steck K.S., Sauer K., Doiron A. (2019). Enzyme-encapsulating polymeric nanoparticles: A potential adjunctive therapy in *Pseudomonas aeruginosa* biofilm-associated infection treatment. Colloids Surf. B Biointerfaces.

[71] Valero-Leal K., Valbuena E., Chacón F., Olivares Y., Castro G., Briñez W. (2010). Patógenos Contagiosos Y Ambientales Aislados De Cuartos Mamarios Con Mastitis Subclínica De Alto Riesgo En Tres Fincas Del Estado Zulia. Rev. Cient. Fac. Cienc. Vet. Univ. Zulia.

[72] Ramalho R., Cunha J., Teixeira P., Gibbs P.A. (2002). Modified *Pseudomonas* agar: New differential medium for the detection/enumeration of *Pseudomonas aeruginosa* in mineral water. J. Microbiol. Methods.

[73] Augusto C., Escobar M., Vanessa L., Murillo R., Soto J.F. (2011). Tecnologías Bioinformáticas para el Análisis de Secuencias de ADN Bioinformatics Technologies for the Analysis of DNA sequences. Sci. Tech..

[74] Limbago B. (2001). M100-S11, Performance standards for antimicrobial susceptibility testing. Clin. Microbiol. Newsl..

[75] Niederman M.S. (2008). Antibiotic Use in the Mechanically Ventilated Patient. Mechanical Ventilation: Clinical Applications and Pathophysiology.

[76] Hudzicki J. (2009). Kirby-Bauer Disk Diffusion Susceptibility Test Protocol Author Information. American Society for Microbiology. https://www.asm.org/Protocols/Kirby-Bauer-Disk-Diffusion-Susceptibility-Test-Pro.

